# Supplementary material for: Mechanisms governing the pioneering and redistribution capabilities of the non-classical pioneer PU.1
Source: Nat Commun. 2020 Jan 21;11:402. doi: 10.1038/s41467-019-13960-2 (PMC6972792; doi:10.1038/s41467-019-13960-2)
Supplement: Supplementary file 7 — Source data [file 41467_2019_13960_MOESM7_ESM.zip › Source_Data/FigureS3/FigureS3J_MotifScanOutput/homerResults/motif8.similar.html]

motif8

## Information for motif8

A
T
C
G
C
G
T
A
T
A
C
G
G
A
T
C
G
C
A
T
G
A
C
T
C
G
T
A
A
T
C
G
G
C
A
T
A
G
T
C
G
T
C
A
  
Reverse Opposite:  

C
A
G
T
T
C
A
G
C
G
T
A
T
A
G
C
G
C
A
T
C
T
G
A
C
G
T
A
C
T
A
G
A
T
G
C
G
C
A
T
T
A
G
C
  

|  |  |
| --- | --- |
| p-value: | 1e-140 |
| log p-value: | -3.229e+02 |
| Information Content per bp: | 1.560 |
| Number of Target Sequences with motif | 4807.0 |
| Percentage of Target Sequences with motif | 36.42% |
| Number of Background Sequences with motif | 9030.4 |
| Percentage of Background Sequences with motif | 24.74% |
| Average Position of motif in Targets | 402.6 +/- 387.6bp |
| Average Position of motif in Background | 317.1 +/- 207.2bp |
| Strand Bias (log2 ratio + to - strand density) | 0.0 |
| Multiplicity (# of sites on avg that occur together) | 1.25 |
| Motif File: | file (matrix) reverse opposite |

### Similar de novo motifs found

|  |  |  |  |  |  |  |  |
| --- | --- | --- | --- | --- | --- | --- | --- |
| Rank | Match Score | Redundant Motif | P-value | log P-value | % of Targets | % of Background | Motif file |
| 1 | 0.852 | G A T C T C A G C T G A G C A T T A C G C G T A A G T C G C A T T C G A C T G A T C A G A T G C | 1e-107 | -247.908756 | 8.24% | 3.27% | motif file (matrix) |
| 2 | 0.778 | A C G T A C G T C G T A A C T G A C G T A G T C C G T A A T G C | 1e-55 | -127.163786 | 5.88% | 2.76% | motif file (matrix) |
